# Supplementary material for: Differences between returns to education in Urban and rural China and its evolution from 1989 to 2019
Source: PLoS One. 2022 Oct 4;17(10):e0274506. doi: 10.1371/journal.pone.0274506 (PMC9531797; doi:10.1371/journal.pone.0274506)
Supplement: S1 Appendix — (DOCX) [file pone.0274506.s001.docx]

**Appendices**

**Table 4. Returns to Education in Urban China**

| Variables | 1989 | 1991 | 1993 | 1995 | 1997 | 2000 |
| --- | --- | --- | --- | --- | --- | --- |
| Edu | 0.0296**^***^**  (0.0034) | 0.0252**^***^**  (0.0031) | 0.0290**^***^**  (0.0046) | 0.0472**^***^**  (0.0013) | 0.0316**^***^**  (0.0039) | 0.0650**^***^**  (0.0058) |
| Exp | 0.0212**^***^**  (0.0046) | 0.0262**^***^** | 0.0383**^***^** | 0.0324**^***^** | 0.0111**^***^** | 0.0114**^**^** |
|  |  | (0.0046) | (0.0049) | (0.0015) | (0.0038) | (0.0065) |
| Exp^2^ | -0.00015 | -0.00023**^**^** | -0.00052**^***^** | -0.00048**^***^** | -0.00005 | -0.00008 |
|  | (0.0001) | (0.00009) | (0.00011) | (0.00003) | (0.00008) | (0.00014) |
| Gender | 0.105**^***^** | 0.156**^***^** | 0.141**^***^** | 0.125**^***^** | 0.085**^***^** | 0.152**^***^** |
|  | (0.0242) | (0.0218) | (0.0260) | (0.0072) | (0.0195) | (0.0300) |
| Cons | 5.786**^***^** | 5.874**^***^** | 5.938^***^ | 6.458**^***^** | 6.465**^***^** | 6.273**^***^** |
|  | (0.068) | (0.060) | (0.075) | (0.028) | (0.064) | (0.107) |
| Province | YES | YES | YES | YES | YES | YES |
| Obs | 1492 | 1375 | 1737 | 12045 | 1547 | 1640 |
| R^2^ | 0.152 | 0.235 | 0.145 | 0.256 | 0.150 | 0.122 |
| Confidence Interval | [0.0226,  0.0366] | [0.0190,  0.0313] | [0.0199,  0.0381] | [0.0446,  0.0498] | [0.0239,  0.0393] | [0.0545,  0.0769] |

***Note***: Numbers in parentheses denote robust standard error；* significant at 10%, ** significant at 5%, *** significant at 1%; Province denote province fixed effect

**Table 4. Returns to Education in Urban China (Continued)**

| Variables | 2002 | 2004 | 2005 | 2006 | 2008 |
| --- | --- | --- | --- | --- | --- |
| Edu | 0.1106**^***^**  (0.0026) | 0.0895^**^  (0.0066) | 0.1055**^***^**  (0.0048) | 0.0795^***^  (0.0050) | 0.1097^***^  (0.0063) |
| Exp | 0.0261**^***^** | 0.0121**^*^** | 0.0084 | 0.0060 | 0.0008 |
|  | (0.0027) | (0.0066) | (0.0057) | (0.0041) | (0.0059) |
| Exp^2^ | -0.00025**^***^** | -0.00001 | -0.00011 | -0.00001 | -0.00001 |
|  | (0.00005) | (0.00014) | (0.00011) | (0.00009) | (0.00012) |
| Gender | 0.246**^***^** | 0.175**^***^** | 0.298**^***^** | 0.293**^***^** | 0.256**^***^** |
|  | (0.0122) | (0.0328) | (0.0252) | (0.0242) | (0.033) |
| Cons | 5.929**^***^** | 6.3348**^***^** | 6.594**^***^** | 6.904**^***^** | 6.734**^***^** |
|  | (0.524) | (0.129) | (0.108) | (0.102) | (0.170) |
| Province | YES | YES | YES | YES | YES |
| Obs | 12304 | 1243 | 3438 | 2721 | 1800 |
| R^2^ | 0.228 | 0.201 | 0.297 | 0.295 | 0.332 |
| Confidence Interval | [0.1054,  0.1158] | [0.0764,  0.1026] | [0.0961,  0.1149] | [0.0697,  0.0891] | [0.0974,  0.1220] |

**Table 4. Returns to Education in Urban China (Continued)**

| Variables | 2009 | 2010 | 2011 | 2012 | 2013 | 2014 |
| --- | --- | --- | --- | --- | --- | --- |
| Edu | 0.0991^***^  (0.0063) | 0.1151**^***^**  (0.0060) | 0.1097**^***^**  (0.0100) | 0.1006**^***^**  (0.0052) | 0.0961**^***^**  (0.0069) | 0.0809**^***^**  (0.0049) |
| Exp | 0.0281^***^ | 0.0201**^***^** | 0.0407**^***^** | 0.0334**^***^** | 0.0361**^***^** | 0.0346**^***^** |
|  | (0.0074) | (0.0058) | (0.0085) | (0.0054) | (0.0056) | (0.0057) |
| Exp^2^ | -0.00041^**^ | -0.00051**^***^** | -0.00090**^***^** | -0.00077**^***^** | -0.00086**^***^** | -0.00066**^***^** |
|  | (0.00016) | (0.00014) | (0.00020) | (0.00012) | (0.00013) | (0.00012) |
| Gender | 0.236**^***^** | 0.289**^***^** | 0.297**^***^** | 0.283**^***^** | 0.302**^***^** | 0.315**^***^** |
|  | (0.0336) | (0.0296) | (0.0482) | (0.0285) | (0.0278) | (0.0283) |
| Cons | 6.541**^***^** | 6.970**^***^** | 7.030**^***^** | 7.275**^***^** | 7.388**^***^** | 6.996**^***^** |
|  | (0.133) | (0.122) | (0.207) | (0.114) | (0.137) | (0.138) |
| Province | YES | YES | YES | YES | YES | YES |
| Obs | 1251 | 1895 | 880 | 2056 | 1810 | 3322 |
| R^2^ | 0.268 | 0.410 | 0.392 | 0.421 | 0.427 | 0.172 |
| Confidence Interval | [0.0866,  0.1115] | [0.1033,  0.1268] | [0.0899,  0.1293] | [0.0905,  0.1108] | [0.0825,  0.1098] | [0.0712,  0.0905] |

**Table 4. Returns to Education in Urban China (Continued)**

| Variables | 2015 | 2016 | 2017 | 2018 | 2019 |
| --- | --- | --- | --- | --- | --- |
| Edu | 0.1053**^***^**  (0.0068) | 0.0858**^***^**  (0.0096) | 0.1126**^***^**  (0.0027) | 0.0852**^***^**  (0.0045) | 0.0984**^***^**  (0.0076) |
| Exp | 0.0239**^***^** | 0.0182**^*^** | 0.0425**^***^** | 0.0268**^***^** | 0.0418**^***^** |
|  | (0.0057) | (0.0095) | (0.0025) | (0.0046) | (0.0088) |
| Exp^2^ | -0.00056**^***^** | -0.00046**^**^** | -0.00088**^***^** | -0.00054**^***^** | -0.00084**^***^** |
|  | (0.00013) | (0.00021) | (0.00006) | (0.00010) | (0.00020) |
| Gender | 0.258**^***^** | 0.325**^***^** | 0.279**^***^** | 0.318**^***^** | 0.360**^***^** |
|  | (0.0328) | (0.0522) | (0.0127) | (0.0235) | (0.0409) |
| Cons | 7.388**^***^** | 7.148**^***^** | 7.014**^***^** | 7.303**^***^** | 7.105**^***^** |
|  | (0.127) | (0.278) | (0.054) | (0.130) | (0.184) |
| Province | YES | YES | YES | YES | YES |
| Obs | 1475 | 1314 | 13003 | 3197 | 1180 |
| R^2^ | 0.378 | 0.199 | 0. 309 | 0.244 | 0.288 |
| Confidence Interval | [0.0920,  0.1186] | [0.0669,  0.1047] | [0.1074,  0.1179] | [0.0762,  0.0942] | [0.0834  0.1136] |

**Table 5. Returns to Education in Rural China**

| Variables | 1989 | 1991 | 1993 | 1995 | 1997 | 2000 |
| --- | --- | --- | --- | --- | --- | --- |
| Edu | 0.0061  (0.0051) | 0.0203^***^  (0.0053) | 0.0008  (0.0086) | 0.0183^**^  (0.0074) | 0.0021  (0.0090) | 0.0171**^*^**  (0.0088) |
| Exp | 0.0258**^***^** | 0.0283^***^ | 0.0044 | 0.0151^**^ | 0.0153**^*^** | 0.0145**^*^** |
|  | (0.0055) | (0.0053) | (0.0076) | (0.0067) | (0.0085) | (0.0084) |
| Exp^2^ | -0.00039**^***^** | -0.00041^***^ | -0.00004 | -0.00028^*^ | -0.00045^**^ | -0.00034**^*^** |
|  | (0.00012) | (0.00012) | (0.00019) | (0.00016) | (0.00020) | (0.00019) |
| Gender | 0. 168^***^ | 0.211**^***^** | 0.110^**^ | 0.147**^***^** | 0.301**^***^** | 0.241**^***^** |
|  | (0.0320) | (0.0284) | (0.0446) | (0.0368) | (0.0511) | (0.0500) |
| Cons | 5.948**^***^** | 5.885**^***^** | 6.521**^***^** | 6.715**^***^** | 6.628**^***^** | 6.896**^***^** |
|  | (0.088) | (0.086) | (0.121) | (0.112) | (0.140) | (0.137) |
| Province | YES | YES | YES | YES | YES | YES |
| Obs | 1388 | 1269 | 622 | 1252 | 612 | 691 |
| R^2^ | 0.086 | 0.176 | 0.076 | 0.088 | 0.119 | 0.103 |
| Confidence Interval | [-0.0038,  0.0160] | [0.0099,  0.0309] | [-0.0161,  0.0178] | [0.0039,  0.0380] | [-0.0156,  0.0199] | [-0.0003,  0.0346] |

**Table 5. Returns to Education in Rural China (Continued)**

| Variables | 2002 | 2004 | 2005 | 2006 | 2008 |
| --- | --- | --- | --- | --- | --- |
| Edu | 0.0586**^***^**  (0.0055) | 0.0345**^***^**  (0.0131) | 0.0500**^***^**  (0.0058) | 0.0232**^***^**  (0.0088) | 0.0571**^***^**  (0.0089) |
| Exp | 0.0138**^***^** | 0.0097 | 0.0127 | 0.0142**^*^** | 0.0298**^***^** |
|  | (0.0040) | (0.0088) | (0.0084) | (0.0076) | (0.0090) |
| Exp^2^ | -0.00052**^**^** | -0.00037**^*^** | -0.00001 | -0.00009 | -0.00025 |
|  | (0.00009) | (0.00021) | (0.00015) | (0.00019) | (0.00017) |
| Gender | 0.154**^***^** | 0.227**^***^** | 0.501**^***^** | 0.399^***^ | 0.451**^***^** |
|  | (0.0262) | (0.0567) | (0.0329) | (0.0417) | (0.0460) |
| Cons | 6.378**^***^** | 7.193**^***^** | 7.209**^***^** | 7.747**^***^** | 7.604**^***^** |
|  | (0.094) | (0.175) | (0.178) | (0.157) | (0.287) |
| Province | YES | YES | YES | YES | YES |
| Obs | 9688 | 401 | 3025 | 1098 | 1439 |
| R^2^ | 0.153 | 0.141 | 0.244 | 0.225 | 0.448 |
| Confidence Interval | [0.0478,  0.0695] | [0.0089,  0.0602] | [0.0386,  0.0614] | [0.0055,  0.0408] | [0.0396,  0.0745] |

**Table 5. Returns to Education in Rural China (Continued)**

| Variables | 2009 | 2010 | 2011 | 2012 | 2013 | 2014 |
| --- | --- | --- | --- | --- | --- | --- |
| Edu | 0.0315**^***^**  (0.0102) | 0.0383**^***^**  (0.0064) | 0.0381**^***^**  (0.0093) | 0.0466**^***^**  (0.0057) | 0.0392**^***^**  (0.0061) | 0.0324**^***^**  (0.0039) |
| Exp | 0.0186**^*^** | 0.0079 | 0.0041 | 0.0034 | 0.0159**^**^** | 0.0405**^***^** |
|  | (0.0095) | (0.0067) | (0.0087) | (0.0064) | (0.0064) | (0.0044) |
| Exp^2^ | -0.00043**^**^** | -0.00023**^*^** | -0.00033**^*^** | -0.00055**^***^** | -0.00074**^***^** | -0.00089**^***^** |
|  | (0.00021) | (0.00013) | (0.00018) | (0.00013) | (0.00013) | (0.00009) |
| Gender | 0.272**^***^** | 0.448**^***^** | 0.576**^***^** | 0.631**^***^** | 0.554**^***^** | 0.456**^***^** |
|  | (0.0508) | (0.0353) | (0.0456) | (0.0338) | (0.0334) | (0.0243) |
| Cons | 7.565**^***^** | 7.878**^***^** | 8.258**^***^** | 7.896^***^ | 8.226**^***^** | 7.652**^***^** |
|  | (0.169) | (0.207) | (0.158) | (0.157) | (0.147) | (0.133) |
| Province | YES | YES | YES | YES | YES | YES |
| Obs | 633 | 2604 | 1454 | 2733 | 2721 | 5783 |
| R^2^ | 0.129 | 0.283 | 0.322 | 0.391 | 0.355 | 0.126 |
| Confidence Interval | [0.0114,  0.0515] | [0.0257,  0.0509] | [0.0200,  0.0563] | [0.0353,  0.0578] | [0.0271,  0.0513] | [0.0247,  0.0399] |

**Table 5. Returns to Education in Rural China (Continued)**

| Variables | 2015 | 2016 | 2017 | 2018 | 2019 |
| --- | --- | --- | --- | --- | --- |
| Edu | 0.0594**^***^**  (0.0060) | 0.0472**^***^**  (0.0055) | 0.0763**^***^**  (0.0023) | 0.0454**^***^**  (0.0037) | 0.0495**^***^**  (0.0094) |
| Exp | 0.0174**^***^** | 0.0397**^***^** | 0.0439**^***^** | 0.0334**^***^** | 0.0739**^***^** |
|  | (0.0066) | (0.0075) | (0.0024) | (0.0044) | (0.0098) |
| Exp^2^ | -0.00071**^***^** | -0.00082**^***^** | -0.00098**^***^** | -0.00081**^***^** | -0.00185**^***^** |
|  | (0.00014) | (0.00016) | (0.00005) | (0.00009) | (0.00020) |
| Gender | 0.477**^***^** | 0.408**^***^** | 0.279**^***^** | 0.487**^***^** | 0.493**^***^** |
|  | (0.034) | (0.0389) | (0.0101) | (0.0235) | (0.0507) |
| Cons | 8.271**^***^** | 7.276**^***^** | 7.543**^***^** | 7.668**^***^** | 7.175**^***^** |
|  | (0.185) | (0.163) | (0.053) | (0.102) | (0.397) |
| Province | YES | YES | YES | YES | YES |
| Obs | 2636 | 3104 | 15757 | 5797 | 1762 |
| R^2^ | 0.359 | 0.098 | 0.220 | 0.160 | 0.197 |
| Confidence Interval | [0.0475  0.0712] | [0.0363,  0.0581] | [0.0718,  0.0806] | [0.0378  0.0529] | [0.0311,  0.0678] |
