# Supplementary material for: Differences between returns to education in Urban and rural China and its evolution from 1989 to 2019
Source: PLoS One. 2022 Oct 4;17(10):e0274506. doi: 10.1371/journal.pone.0274506 (PMC9531797; doi:10.1371/journal.pone.0274506)
Supplement: Attachment — Submitted filename: PLOS ONE Comments.docx [file pone.0274506.s002.docx]

PONE-D-22-03043

**The Difference Between Returns to Education in Urban and Rural China and Its**

**Dynamic Changes From 1989 to 2019**

The authors estimated the differences in returns to education in urban and rural China over the past 30 years and analyzed the underlying causes. This is a very important and interesting topic relevant to many contexts. However, I do have some comments, which hopefully will be helpful in improving the paper.

1. Introduction: Returns to education is an important topic, but the introduction is not compelling enough to highlight its importance and why it matters even more in China.
2. Literature review: First of all, my understanding is that literature review should be a critical review rather than a list of citations stacking on top of each other. In the meantime, I would suggest that the authors cite some recent studies on the returns to education. For example, the one by Liu and her coauthors titled “The returns to education in rural China: Some new estimates.”
3. Data: I would also suggest the authors to provide more detailed introductions about the data used in the study, especially when the authors declare their biggest contribution is to integrate various micro databases. BTW, what do CHNS, CGSS, CULS, and CHIP stand for? Are they all nationally representative datasets? Are they panel or pooled OLS? How did you combine them together? Please note the data sources for the tables and figures.
4. Measurement:
   a) Did you take price into consideration when measuring income? I think it is necessary to do so given such a long time span studied.
   b) BTW, how did you measure marketization in your empirical analysis?
   c) Table 3: Please clarify whether they measure the same thing? Different indexes constructed by different scholars, unless using the same method and data, otherwise might be apples and oranges.
5. Methodology:
   a) The authors should clarify the rationale for dividing the urban and rural subsamples, whether by *hukou* or by geographic location. Since China has a large number of migrant rural workers living in urban areas, if the division is by geographic area, the increased returns to education of those population are also attributed to urban returns to education, which may result in an underestimation of rural returns to education. Besides, the authors should test whether the differences of returns to education between urban and rural are significant.
   b) IV: Spouse’s education level does not necessarily satisfy the exogeneity of the instrumental variable. The authors need to further justify why some already widely used instrumental variables, such as the Compulsory Education Law (Fang et al., 2012) or the Prohibition of Using Child Labor (Xie & Mo, 2014), are not adopted. In addition, appropriate models for the IV estimation need to be specified.
   c) I was wondering what the results look like if you control for the marital status of individuals and their employment sectors.
   d) How do your OLS and IV results compare with each other?
6. Mechanism: This paper explores the mechanism relatively well. However, it is essentially narrative hypotheses/arguments. Please do provide empirical evidence, whenever data available for quantitative analyses, to test your hypotheses, and thus to support your arguments.
7. Presentation:
   a) Please transpose Table 1 so that it looks more compact and neat.
   b) Table 2, Please report all results or at least provide results for other variables in appendix.
   c) Figure 1: Please also report the confidence interval.
8. The writing, including the explanations of data, variable definition, models, needs to be improved. In addition, the vertical axes in the figures all lack units.
9. Others: What do you mean by inflection point?
