# Supplementary material for: Differences between returns to education in Urban and rural China and its evolution from 1989 to 2019
Source: PLoS One. 2022 Oct 4;17(10):e0274506. doi: 10.1371/journal.pone.0274506 (PMC9531797; doi:10.1371/journal.pone.0274506)
Supplement: Attachment — Submitted filename: Response to Reviewers.docx [file pone.0274506.s003.docx]

**Response to Reviewers**

Thanks to the valuable suggestions from Academic Editor and Reviewers on the manuscript. We have tried our best to revise the paper according to suggestions. Now for you to propose a brief reply:

**Academic Editor**

1. make the introduction more compelling, focusing on the relevance of the differences in returns to education in China (R1)

**Answer**: We have revised the introduction to highlight the importance of the topic, the returns to education, in China.

2. Please consider reorganizing your literature review, trying to be more critical and thus more informative (R1)

**Answer**: We have revised the literature review to make it more critical and provide more information.

3. describe in detail the data sources you use, their representativeness, and justify all your choices (i.e. why you use some waves and not other). Please provide the reader with detailed information also about how you merged the data, in order to increase the replicability of your results. You can also devote one or more appendixes to this very important issue, if you prefer. (R1 and R2)

**Answer**: We have stated the sources of each data in this paper. We primarily used data from CGSS, and added data from other databases for other years that were included in the long time span. Although different survey datasets have different sampling strategies and weights, the sampling of each micro-data is nationally representative. Therefore, in the end, we combined multiple micro-data for the dynamic analysis. We used the data of each year separately, and we have attached the complete results in the appendixes.

4. I agree with both reviewers that the data and methodology sections require some rewriting. I suggest considering inflation in the definition of your dependent variable and provide a discussion of identification threats your model faces (endogeneity), and how you solve them (with an IV approach), introducing a new econometric model directly in section 2.1. (R1 and R2)

**Answer**: As suggested by Academic Editor, we have revised the data and methodology. The annual income has been adjusted according to the Consumer Price Index (CPI) to eliminate the influence of inflation. At the same time, in Section 2.1, we introduced the IV model. We have also further explained and analyzed the selection of instrumental variables in the manuscript.

5. I agree with Reviewer #1 that your empirical strategy (i.e., IV) does not allow to take into account properly potential endogeneity, as the instruments you use may have a direct effect on the dependent variable and hence the results cannot be interpreted as causal relationships. For more details, please refer to the comments of the Reviewers reported below. R1 suggests two alternative instruments that are worth exploring.

**Answer**: The educational level of the spouse may not be the optimal choice for the instrumental variable. However, due to the long time span of the data used in this study, it is difficult to apply the instrumental variables based on policy implementation to the data of each year. The educational level of the spouse is better than other instrumental variables. Therefore, in this study, the educational level of the spouse was selected as the instrumental variable for the robustness test. In addition, this paper focuses on the long-term dynamic changes in the returns to education rather than emphasizing the level of the returns to education at a certain time point. If the correlation coefficient between the unobservable ability factor and the educational level and income level has a fixed or approximately fixed time trend, the unobservable ability factor does not affect the analysis of the changing trend of the returns to education. Therefore, the educational level of the spouse was still used as the instrumental variable for the robustness test.

6. If it possible, I encourage you to provide some empirical support, even suggestive, to the mechanisms you describe. (R1)

**Answer**: In the cause analysis of the dynamic changes in returns to education, as it is greatly limited by data, we only performed qualitative analysis rather than quantitative analysis. This study covers a very long time span, and hence, it is difficult for us to find suitable data for empirical analysis. Of course, this is also a shortcoming of this paper, and we hope to solve this problem in future research. However, in this paper, we tried our best to explain the reasons for the dynamic changes in return to education in China through qualitative analysis.

**Reviewer #1**

1. Introduction: Returns to education is an important topic, but the introduction is not compelling enough to highlight its importance and why it matters even more in China.

**Answer**: We have revised the introduction to highlight the importance of the topic, the returns to education, in China.

2. Literature review: First of all, my understanding is that literature review should be a critical review rather than a list of citations stacking on top of each other. In the meantime, I would suggest that the authors cite some recent studies on the returns to education. For example, the one by Liu and her coauthors titled “The returns to education in rural China: Some new estimates.”

**Answer**: We have revised the literature review to make it more critical and cited some recent research on returns to education.

3. Data: I would also suggest the authors to provide more detailed introductions about the data used in the study, especially when the authors declare their biggest contribution is to integrate various micro databases. BTW, what do CHNS, CGSS, CULS, and CHIP stand for? Are they all nationally representative datasets? Are they panel or pooled OLS? How did you combine them together? Please note the data sources for the tables and figures.

**Answer**: We have more clearly stated the sources of the data in this paper. In particular, they are China Health and Nutrition Survey (CHNS), Chinese Household Income Project Survey (CHIP), Chinese General Social Survey (CGSS), China Family Panel Studies (CFPS), China Household Finance Survey (CHFS), and Chinese Social Survey (CSS). Some data used are panel data, while some are mixed cross-sectional data. We used the data of each year separately, and we have indicated the data sources for the tables and figures in this paper.

4. Measurement:
   a) Did you take price into consideration when measuring income? I think it is necessary to do so given such a long time span studied.
   b) BTW, how did you measure marketization in your empirical analysis?
   c) Table 3: Please clarify whether they measure the same thing? Different indexes constructed by different scholars, unless using the same method and data, otherwise might be apples and oranges.

**Answer**: As suggested, we have adjusted the annual income according to the Consumer Price Index (CPI) to eliminate the influence of inflation. We did not use the marketization index in our empirical analysis. However, in the cause analysis of dynamic changes in the returns to education, we used the marketization index and the marketization index data of existing research to explain the problem. Table 3 gives two marketization indexes. The construction principles of the two marketization indexes are different, and they are not directly comparable, but we can still see the labor market process in a certain period. Therefore, we combined the two marketization indexes and roughly inferred the changing characteristics of the degree of labor marketization in China.

5. Methodology:
   a) The authors should clarify the rationale for dividing the urban and rural subsamples, whether by *hukou* or by geographic location. Since China has a large number of migrant rural workers living in urban areas, if the division is by geographic area, the increased returns to education of those population are also attributed to urban returns to education, which may result in an underestimation of rural returns to education. Besides, the authors should test whether the differences of returns to education between urban and rural are significant.
   b) IV: Spouse’s educational level does not necessarily satisfy the exogeneity of the instrumental variable. The authors need to further justify why some already widely used instrumental variables, such as the Compulsory Education Law (Fang et al., 2012) or the Prohibition of Using Child Labor (Xie & Mo, 2014), are not adopted. In addition, appropriate models for the IV estimation need to be specified.
   c) I was wondering what the results look like if you control for the marital status of individuals and their employment sectors.
   d) How do your OLS and IV results compare with each other?

**Answer**: The urban and rural samples in this paper are divided by *hukou*, and the reasons for the division are provided in this paper. As suggested, in this paper, we tested whether the difference between returns to education in urban and rural China is significant. The educational level of the spouse may not be the best choice for the instrumental variable. However, due to the long time span of the data used in this paper, it is difficult to apply the instrumental variable based on policy implementation to the data of each year, and the educational level of the spouse is relatively better than other instrumental variables. Therefore, in this paper, the educational level of the spouse was selected as the instrumental variable for the robustness test. Besides, this paper focuses on the long-term dynamic changes in the returns to education rather than emphasizing the level of the return to education at a certain time point. If the correlation coefficient between the unobservable ability factor and the educational level and income level has a fixed or approximately fixed time trend, the unobservable ability factor does not affect the analysis of the changing trend of the returns to education (Zhong and Liu, 2007). Therefore, the educational level of the spouse was still used as the instrumental variable for the robustness test. We also introduced the IV model in this paper. Since the data of some years have no individual characteristic index, such as marine status and employment sectors, or have no family characteristic variable to maintain the uniformity of the control variables of all years, we selected only the gender as the control variable. In the results of this paper, the estimation of IV is greater than that of OLS.

6. Mechanism: This paper explores the mechanism relatively well. However, it is essentially narrative hypotheses/arguments. Please do provide empirical evidence, whenever data available for quantitative analyses, to test your hypotheses, and thus to support your arguments.

**Answer**: In the cause analysis of the dynamic changes in returns to education, as it is greatly limited by data, we only performed qualitative analysis rather than quantitative analysis. This study covers a very long time span, and hence, it is difficult for us to find suitable data for empirical analysis. Of course, this is also a shortcoming of this paper, and we hope to solve this problem in future research. However, in this paper, we tried our best to explain the reasons for the dynamic changes in return to education in China through qualitative analysis.

7. Presentation:
   a) Please transpose Table 1 so that it looks more compact and neat.
   b) Table 2, Please report all results or at least provide results for other variables in appendix.
   c) Figure 1: Please also report the confidence interval.

**Answer**: Table 1 has been adjusted, and the complete results are shown in the appendixes (Tables 4 and 5). We have not found a way to put the confidence interval in Fig 1, but we have put the confidence interval in the tables in the appendixes (Tables 4 and 5).

8. The writing, including the explanations of data, variable definition, models, needs to be improved. In addition, the vertical axes in the figures all lack units.

**Answer**: We have refined contents including the explanations of data, variable definition, and models. Units are also added to the vertical axes of the figures.

9. Others: What do you mean by inflection point?

**Answer**：Inflection point refers to the turning point of returns to education from high to low.

**Reviewer #2**

1. My first primary concern is related to the combination of the multiple datasets from different institutions using different methods of data collection. In Section 2.2 Data sources, the authors claimed that they used China Health and Nutrition Survey (CHNS) for 1989, 1991, 1993, 1997, 2000, 2004, and 2009. As far as I know, up to now, CHNS has made 10 waves available, including 1989, 1991, 1993, 1997, 2000, 2004, 2006, 2009, 2011, and 2015. So, why the authors did not use the data from 2006, 2011, and 2015? Similarly, for the Chinese General Social Survey (CGSS), there are additional waves already coming out, CGSS 2015 and 2017. I understand that there are several overlapping years among the major social survey datasets, but please provide a full explanation and rationale of why choosing one is not the other. The starting point is to offer readers the full names and more details of each dataset so that for some of the dataset selections we would understand that they were based on the data availability. Moreover, most of the datasets, such as CGSS, are cross-sectional, but CFPS is a longitudinal dataset. Different survey datasets have different sampling strategies and weighting. More discussions are needed to see how authors made a balance among different datasets for consistent analysis. Also, in terms of the urban vs. rural, there is a potential concern as the study spans the past decades. As China has experienced substantial development of urbanization since the economic reform in the 1980s, what was considered rural in the 1990s may have become urban areas in the 2010s.

**Answer**: We used so much data because each year’s data were used separately. The main purpose of this paper is to analyze the dynamic changes in the difference between returns to education in urban and rural China, thus requiring data over several years. However, many micro-data of a single source do not have data of consecutive years, and span a relatively short period of time. To have a long time span and continuous data, we combined multiple micro-data. We primarily used data from CGSS, and added data from other databases for other years that were included in the long time span. Although different survey datasets have different sampling strategies and weights, the sampling of each micro-data is nationally representative. Therefore, in the end, we combined multiple micro-data for the dynamic analysis. Of course, as you mentioned, different micro-databases have different sampling methods, errors in the sampling process, and collaboration of respondents. Then, when the dynamic analysis is made combined with different databases, there may be a risk of biased conclusion, which is also the shortcoming of this paper. However, such a practice is an innovation we attempted. If, in the future, we need to find higher-quality unified micro survey data, we will analyze dynamic changes in the returns to education again. In addition, the urban and rural samples in this paper are divided by *hukou*, and the reasons for the division are provided in this paper.

2. The second major point is to related to variables and analysis. In 2.3 Description of variables, it says that the dependent variable is annual income. First, based on the skewed distribution of annual income, the variable is supposed to be logged. Second, the variable of annual income needs to add CPI-adjusted to take inflation over years into consideration. As for the independent variables, age may need to add a square term as the relationship between age and income may not be linear. Moreover, the variable work experience is based on age minus years of education. When including education, age, and work experience, I suspect that there may be issues of multicollinearity. Please recheck the models. In addition, as the authors demonstrated in the discussions on how the hukou system plays a significant role, then why hukou is not a control variable in the models? Here, I recommend a relevant article: The influence of hukou and college education in China’s labour market, by Xiao and Bian (2017) published at Urban Studies.

**Answer**: The annual income in this paper has been adjusted according to the Consumer Price Index (CPI) to eliminate the influence of inflation. Work experience, rather than age, is used as the control variable in this paper. Drawing on the practice of most literatures, “age of an individual–years of education–6” is used to represent work experience. We also controlled the quadratic term of work experience. *Hukou* cannot be controlled as it is used as the basis for the division of urban and rural samples in this paper.

3. Additional minor points and suggestions the authors may wish to consider is the elegancy of tables. For example, in Table 2, it is better to keep two decimals rather than four which will reduce the length of the numbers. For the column of difference, instead of presenting the exact numbers, it’s better to use asterisks to indicate whether they are significant (* p<.05, ** p<.01, *** p<.001).

**Answer**: We made improvements on the presentation of Table 2 as suggested. However, some values of the results shown in Table 2 are small, and cannot be clearly indicated if they had only two decimal places. Therefore, results in Table 2 remained to be in four decimal places. In addition, whether the difference between returns to education in urban and rural China is significant is also tested in Table 2.
