# Supplementary material for: Differences between returns to education in Urban and rural China and its evolution from 1989 to 2019
Source: PLoS One. 2022 Oct 4;17(10):e0274506. doi: 10.1371/journal.pone.0274506 (PMC9531797; doi:10.1371/journal.pone.0274506)
Supplement: Attachment — Submitted filename: Comments Plos R1.docx [file pone.0274506.s004.docx]

Review PONE-D-22-03043R1

**Comments to Editor**

Overall, the revised version has improved and addressed most of concerns I raised last time but still, it needs some minor revisions before it is acceptable for publication. In the Comments to the Author, I summarize several suggestions for the authors.

**Comments to the Author**

Overview and general recommendation:

It is great to see the revised manuscript has addressed most of the comments I raised in a previous round of review. But, there are additional suggestions for minor revisions:

1. The authors added a footnote of the five of the six dataset they used in the analysis, but it is essential to provide more than a simply web link to the dataset but more detailed introduction to the datasets. What are the full names of these datasets? Which institutions designed and collected the data? Are they cross-sectional or longitudinal? And why they are reliable data sources?
2. The authors added a line to indicate that CPI at provincial level has been considered, but it is necessary here to add a footnote of data source and a brief discussion how CPI was adjusted.
3. The authors claimed that “the division of samples is conducted based on respondent’s registered residence, that is, whether they reside in the urban area or the rural area”. This statement is confusing and misleading because where their hukou is registered can be different from where they actually reside in. Millions of workers from rural areas holding rural hukou are working as migrants in cities without their hukou registered in urban areas. If the authors intend to compare the Urban-Rural division based on geographical location, then it is better to divide the sample based on where the respondents reside in and include where their hukou registered (urban or rural) as a control variable.
4. For the section exploring reasons for the dynamic changes of returns to education, they are more like hypotheses with any empirical evidences to prove. I would suggest the authors either trying to find relevant literature to support the hypotheses or selecting a few years that data is available to testify the mechanisms you hypothesized.
5. Add a paragraph or two to address the limitations of this study and provide future research directions. For example, it may be impossible for you to testify all your hypotheses on the mechanisms, then leave them to future researchers to explore.
