# Supplementary material for: Differences between returns to education in Urban and rural China and its evolution from 1989 to 2019
Source: PLoS One. 2022 Oct 4;17(10):e0274506. doi: 10.1371/journal.pone.0274506 (PMC9531797; doi:10.1371/journal.pone.0274506)
Supplement: Attachment — Submitted filename: Response to Reviewers.docx [file pone.0274506.s005.docx]

**Response to Reviewers**

Thanks to the valuable suggestions from Academic Editor and Reviewers on the manuscript. We attach great importance to these suggestions and carefully revise the paper according to the suggestions. Then we asked language experts in this field to polish the manuscript. In the “Revised Manuscript with Track Changes”, **Blue** is the revision trace, and **Red** is the polishing trace. Now we will reply to academic editor and reviewers.

**Academic Editor**

1. I believe that more discussion should be added to the methodology section, where you added the IV model. You should explain to the reader why an alternative approach is needed: why is Edu endogenous? What are the assumptions Z needs to satisfy in order to be a “good instrument”? Are they satisfied? Please, remember that IV is a Local Average Treatment Effect, who are the compliers in your samples? These points should be addressed in the methodological section. In this version of the paper, part of the discussion about the endogeneity is reported in the robustness section, please move the discussion in the methodology section.

**Answer**: We have moved the discussion of endogeneity in the methodology section and provided further explanation of endogeneity and IV, including why is Edu endogenous? What are the assumptions Z needs to satisfy in order to be a “good instrument”? Do the tool variables selected in this article satisfy these assumptions? And who are the compliers in your samples?

2. Rather than talking about “dynamic changes”, I suggest using “evolution/changes over time”, even in the title. To me, and I believe to applied economists in general, the term “dynamic” refers to the study of how today level of a variable depends on the level it had yesterday. Empirically this is assessed using dynamic econometrics models, which often include the lag of the dependent variable. You rather use repeated cross-sections and thus do not model the dynamic in returns to education econometrically.

**Answer**: As you suggested, we have replaced all "dynamic changes" with "evolution" in the paper, including in the title.

3. Please add some confidence intervals to your Figures, they will help a reader in assessing the evolution over time of your coefficients.

**Answer**: We have added confidence intervals in Figures 1 and 2。

4. I do agree with reviewer 1 that you should support your reasoning in the “Reasons for the dynamic changes of returns to education” section with some indirect evidence or references to the literature. You do not need to produce original evidence, but you should ground your reasoning on other research, otherwise it appears to be speculation. To illustrate, a sentence like "The reform of the household registration system mainly has a greater influence on returns to education in rural China." is a strong one. Who say that this reform had a great influence? You? Somebody else? If so, you should quote the research. You go on saying: " Returns to education in rural China is low mainly due to the institutional segmentation caused by the unique household registration system in China, which results in the low efficiency of labor distribution and the non-competitive labor market with low mobility. " This is an even stronger affirmation: you are establishing a causal link from the household registration system to low returns to education. Is there evidence of the causal link? If so you should quote the papers/literature/research, otherwise, you should ton down your sentence and make is suggestive. As you see, this section needs a substantial rewriting in order to be published, because, in its current form, it contains some affirmations that are not supported by direct or indirect evidence.

**Answer**: As suggested, we have supported the reasoning in the "Reasons for the Dynamic Changes of Returns to education" section with some references to the literature.

5. As suggested also by reviewer 1, please explain in the paper the limitations of your paper.

**Answer**: At the end of the "Conclusion and discussion" section, we have explained the research limitations of this paper.

6. Finally, as suggested also by reviewer 2, the paper requires a thorough professional English editing to meet PLOS ONE publication criteria, since the language is not always clear: please seek independent editorial help before submitting a revision.

**Answer:** We have sought independent editorial help to improve the language and overall content of this paper.

7. Please review your reference list to ensure that it is complete and correct. If you have cited papers that have been retracted, please include the rationale for doing so in the manuscript text, or remove these references and replace them with relevant current references. Any changes to the reference list should be mentioned in the rebuttal letter that accompanies your revised manuscript. If you need to cite a retracted article, indicate the article’s retracted status in the References list and also include a citation and full reference for the retraction notice.

**Answer:** Since we used some literature to support reasoning in the " Reasons for the dynamic changes of returns to education " section, we added the literature to the references, including references 37, 44, 45, 50, 51, 52, 53, 56, 57, 59, 64, 65, 66, 67.

**Reviewer #2**

1. The authors added a footnote of the five of the six dataset they used in the analysis, but it is essential to provide more than a simply web link to the dataset but more detailed introduction to the datasets. What are the full names of these datasets? Which institutions designed and collected the data? Are they cross-sectional or longitudinal? And why they are reliable data sources?

**Answer**: We have further introduced 6 data in footnotes, including full names of these datasets, institutions designed and collected the data, cross-sectional or longitudinal data and why they are reliable data sources.

2. The authors added a line to indicate that CPI at provincial level has been considered, but it is necessary here to add a footnote of data source and a brief discussion how CPI was adjusted.

**Answer**: We have added a footnote of data source and a brief discussion how CPI was adjusted.

3. The authors claimed that “the division of samples is conducted based on respondent’s registered residence, that is, whether they reside in the urban area or the rural area”. This statement is confusing and misleading because where their hukou is registered can be different from where they actually reside in. Millions of workers from rural areas holding rural hukou are working as migrants in cities without their hukou registered in urban areas. If the authors intend to compare the Urban-Rural division based on geographical location, then it is better to divide the sample based on where the respondents reside in and include where their hukou registered (urban or rural) as a control variable..

**Answer**: Our sample is divided by respondent’s registered residence, and we apologize for the misunderstanding caused by the second half of this sentence, which we did not explain clearly. Therefore, in the paper, we have removed the second half of the sentence to eliminate the misunderstanding.

4. For the section exploring reasons for the dynamic changes of returns to education, they are more like hypotheses with any empirical evidences to prove. I would suggest the authors either trying to find relevant literature to support the hypotheses or selecting a few years that data is available to testify the mechanisms you hypothesized.

**Answer**: For the section exploring reasons for the dynamic changes of returns to education, We have supported this part of the reasoning through with some references to the literature.

5. Add a paragraph or two to address the limitations of this study and provide future research directions. For example, it may be impossible for you to testify all your hypotheses on the mechanisms, then leave them to future researchers to explore.

**Answer**: At the end of the "Conclusion and discussion" section, we have addressed the research limitations and provide future research directions of this paper.

**Reviewer #3**

1. The authors have well addressed the reviewers' comments and made corresponding revisions, and the current version of the manuscript has basically met the standard for publication. To make some further improvements, there are a few suggestions. First, the manuscirpt needs language editing to improve the language and overall content. Second, the conlusion section could be further improved by adding content on the contributions and limitations of the study.

**Answer**: We have sought independent editorial help to improve the language and overall content of this paper. At the end of the "Conclusion and discussion" section, we have added content on the contributions, limitations of the study and future research directions.
